# Sjögren’s Hands-On Practice Exchange (SHAPE): a qualitative, expert opinion project in Sjögren’s disease clinical practice

**DOI:** 10.1186/s41927-025-00606-8

**Published:** 2025-12-20

**Authors:** Emily Gregg, Charlotte Graham, Deborah Watkins, Viviam Canon Garcia, Rachael McCool

**Affiliations:** 1 York Health Economics Consortium, Enterprise House, University of York, Heslington, YO10 5NQ UK; 2 Novartis Pharma AG, Basel, CH-4056 Switzerland

**Keywords:** Sjögren’s disease, Rheumatic disease, Qualitative research, Expert opinion

## Abstract

**Background:**

Sjögren’s disease (SjD) is a multifaceted, systemic autoimmune disease with substantial clinical heterogeneity. The objective of this study was to conduct a qualitative expert opinion exercise to explore how SjD is assessed, evaluated and managed in international clinical practice.

**Methods:**

A qualitative research design was used to elicit the expert opinion of 8 clinicians. Two researchers interviewed each clinician individually. The results of the interviews were synthesised using thematic analysis, and draft definitions of key terms were prepared. A group workshop was held to discuss/validate the interview results and refine the working definitions.

**Results:**

The clinicians had extensive experience in managing SjD from different countries. Seven topics emerged as major themes: disease classification, disease activity, relevant subpopulations, disease severity, disease progression, disease remission, and unmet needs. For 4 of these topics, there was no consensus – particularly regarding definitions that could be used in a clinical setting. The terms “systemic involvement” and “extra-glandular symptoms” have similar meanings but lack consistent application between clinicians. There was a lack of consensus on what “severity” refers to – whether it is severity of disease damage, the nature of disease manifestations, the potential for irreversible damage, or level of symptoms. There were also conflicting opinions regarding whether patient perspectives should be incorporated in key definitions.

**Conclusion:**

There is currently a lack of standard definitions specifically for daily practice, which may contribute to high variability in clinical assessment and management. The definitions proposed in this study represent initial working concepts and are intended as a first step to promote further discussion among SjD experts to facilitate a more unified routine evaluation of patients.

**Clinical trial number:**

Not applicable.

**Supplementary Information:**

The online version contains supplementary material available at 10.1186/s41927-025-00606-8.

## Background

Sjögren’s disease (SjD) is a chronic autoimmune disease that affects exocrine glands, impairing their function and reducing the secretion of fluids. Impairment of the lacrimal and salivary glands leads to dryness of the eyes and mouth, respectively – a hallmark of the disease [1]. However, defining SjD simply by symptoms of dryness undermines the systemic and multifaceted nature of the disease [2]. SjD can affect a wide range of organs (e.g. lungs, kidney) and systems (e.g. haematological, musculoskeletal, gastrointestinal and nervous system), with patients experiencing a highly variable symptom burden including fatigue and pain [2–4]. Consequently, SjD is a disabling condition with a considerable impact on quality of life and substantial clinical heterogeneity [3].

The risk of developing a second autoimmune disorder (such as rheumatoid arthritis and lupus) is particularly high among people with SjD [5]. This has been historically referred to as ‘secondary’ SjD; however, this nomenclature is now viewed as inaccurate because there is insufficient clinical and biological evidence to distinguish between primary (occurs without another systemic autoimmune disease) and secondary SjD [6–9]. There is a paucity of epidemiological data for SjD, but a 2024 systematic review estimated that the global prevalence of primary SjD is between 22 and 770 per 100,000 persons, with women having the highest prevalence [10].

There are a number of validated tools to assess disease activity and symptoms (e.g. ESSDAI, ESSPRI), but these indexes were designed for research purposes, and a consensus on important disease definitions for use in day-to-day clinical practice has not yet been established. There are currently no therapies specifically approved for SjD. Therefore, treatment primarily focuses on symptom management – with therapeutic decisions guided by disease guidelines (e.g. EULAR [11]) and clinical presentation [2]. Many treatments are adopted from other rheumatic indications and have limited efficacy in preventing further damage to the salivary glands and other organs [3].

The Sjögren’s Hands-On Practice Exchange (SHAPE) project facilitated a reflection exercise to form a starting point for future research in the field. Specifically, SHAPE aimed to identify the commonalities and divergences in international clinical practice. The project did not aim to define or validate any new clinical trial outcomes or tools, nor to produce clinical guidelines, which are being explored in other initiatives (e.g. NECESSITY, OMERACT) and are led by experts in the disease [12, 13]. Instead, SHAPE complements these existing frameworks by focusing on day-to-day clinical practice and definitions, as opposed to clinical trial endpoints. The objectives of SHAPE were:

(1) To conduct an expert opinion exercise to explore how SjD is managed, assessed and evaluated in clinical practice around the world.

(2) To identify and discuss current challenges in clinical practice and to suggest potential practical approaches for evaluating disease activity, disease severity, classifying patients with SjD, and establishing treatment goals in SjD.

(3) To share the key learnings with a global audience to encourage further discussion and collaboration in this area.

## Methods

SHAPE used a qualitative research design to elicit the expert opinion of 8 clinicians. The clinicians were recruited using purposive sampling, with the sample size based on reaching information saturation and estimated from similar qualitative studies [14–16]. The recruitment criteria for participating clinicians were as follows: the clinicians had to have relevant rheumatology experience on the diagnosis and management of SjD, be fluent in English, and be from a country different to the other participating clinicians. Prospective clinicians were contacted from a shortlist compiled by the study’s sponsor. Ethical approval was obtained from the Health Sciences’ Research Governance Committee at the University of York (approval ID: HSRGC/2024/646/E).

Selected experts were sent an email invitation and information sheet (Supplementary Materials pages 1 to 8) outlining the purpose of the project. Those who agreed to participate signed a written consent form and provided consent for publication. The clinicians received an honorarium for participating based on the fair market value in their country.

### Topic guide

The key areas for discussion in the interviews were detailed in a topic guide (Supplementary Materials pages 9 to 19). This was informed by pragmatic desk-based research to identify recent guidelines and relevant published studies. Before the interviews, the topic guide was piloted on a rheumatologist unrelated to the project.

### Individual interviews

Each interview was scheduled for 60 min and conducted via Zoom between October and November 2024. Two researchers conducted the interviews; one led the interview while the other took notes. The researchers (EG, CG, DW) were trained to minimise common biases during the interviews, including researcher, confirmation, and sampling bias [17]. Two authors (RM and VCG) did not attend the interviews. No full transcripts were produced. Instead, each clinician was offered the opportunity to check their interview summary for accuracy.

Thematic analysis of the interview summaries was conducted by two researchers and validated by a third. Once themes were extracted and agreed, a qualitative synthesis of the themes was conducted, and draft definitions of some key terms were prepared to inform the workshop discussions.

### Workshop

A 3-hour workshop was held with the clinicians in January 2025. This was used to discuss/validate the interview results, to reach some consensus on key concepts for day-to-day clinical practice, and to refine the definitions drafted following the interviews. EG and CG and RM led the workshop, VCG observed the workshop, and DW did not attend.

During the workshop, expert opinions were collected through prompted discussion points, polls, and unprompted conversations. Clinicians were asked to rate their agreement with the draft definitions using a 5-point Likert scale: strongly disagree, disagree, neutral, agree, strongly agree.

One clinician was unable to attend the workshop; they participated by reviewing the workshop slides and responding to the discussion points/polls via email. The results of the workshop were consolidated into a detailed minutes document, including any suggested changes to the definitions. Formal agreement on the revised definitions in the minutes was not measured. The minutes were shared with all 8 participants to check for accuracy.

## Results

Eight clinicians participated in this project. All clinicians were rheumatologists experienced in diagnosing and managing SjD, with a median experience of 24 years (range: 16 to 35 years). Each clinician was based in a different country, including the United States, the United Kingdom (UK), France, Italy, Germany, Australia, China, and Japan. Seven topics emerged as major themes: disease classification, disease activity, relevant subpopulations, disease severity, disease progression, disease remission, and unmet needs. Anonymised quotes are presented to provide evidence for these themes.

### Disease classification

In SjD, it is important to identify and record whether there are any concurrent autoimmune or connective tissue disorders (e.g. lupus or rheumatoid arthritis) because this may change the diagnostic/treatment approach. Most of the interviewed clinicians do not classify SjD using primary/secondary terminology and have alternative ways of distinguishing this, especially to replace “secondary” SjD. For example, one expert prefers “associated with” because “secondary” makes the disease sound less severe.

The interviewed clinicians may also classify people as having “systemic involvement” or “extra-glandular symptoms” – terms that have similar meanings but lack consistent application. Three of the experts exclusively use “systemic”, two clinicians exclusively use “extra-glandular”, and two clinicians use both terms interchangeably (one clinician did not respond). One of the experts who prefers “systemic” commented that extra-glandular is “too vague and potentially confusing.” For example, parotid gland swelling is both systemic and glandular.

### Disease activity

During the interviews, the clinicians reported different ways of defining and exploring disease activity in daily practice. There is also variation in the use of disease indexes (such as the ESSDAI [18] and clinical ESSDAI [clinESSDAI] [19]) in follow-up visits and appointments (which also vary in frequency). Three clinicians stated that they **do not** use disease indexes, and 5 clinicians stated that they **do** use (at least) one index. One clinician noted that the indexes are “difficult to use in practice due to the number of items to remember, and all items can’t be analysed.”

The most frequently used index is the ESSDAI. However, the interviewed experts reported several limitations of the ESSDAI, including that it does not include a gastrointestinal or hepatic domain, it is difficult to measure improvement in the neurological domain during day-to-day clinical practice, it is time consuming to complete in a clinical appointment, and it is a composite score. One clinician added that the “ESSDAI only measures certain domains, and the ESSPRI/ESSDAI correlate poorly.” Despite these concerns, the interviewed clinicians noted that the ESSDAI is the most useful tool that is currently available.

There was an interesting discussion about whether inflammation should be included in a definition of active disease. While the majority of interviewed experts agreed that it **should** be included in some form, there was disagreement regarding the tests used to measure inflammation. The tests most frequently mentioned were erythrocyte sedimentation rate (ESR) and C-reactive protein, but tests for certain cytokines (BAFF, sCD25, IL-6, INF-a) and Th cells (Th1, Th2, Th17, Tfh, B) were also raised. There were conflicting views on whether patient perspective and impact on quality of life (e.g. ESSPRI score, short-form 36 [SF-36], EuroQol 5 Dimensions [EQ-5D]) should be included in a definition for active disease.

A working definition for active disease is presented in the ‘Suggested Definitions’ section of the Results. During the workshop, the clinicians agreed that the definition should be expanded in the future to state ways in which active disease can be measured in daily practice. One clinician stated that guidance on the frequency of assessment for people with SjD would also be useful.

### Subpopulations

Some of the interviewed clinicians reported that people with SjD are classified into subpopulations because of the disease’s heterogeneity in clinical manifestations. Other clinicians reported that while these subpopulations may be observed, no strict categorisation into subpopulations is followed: “people aren’t placed into categories […] because it isn’t necessarily helpful for decision making in clinical practice.”

Subpopulations that were reported as ‘very important’ or ‘somewhat important’ were: risk of developing lymphoma, type of manifestation (e.g. dryness-only symptoms, systemic involvement, organ involvement), age at onset, severity, disease activity, and sex. All of the participating clinicians reported that they identify people at greater risk of developing lymphoma and follow this cohort carefully, perhaps with more regular appointments and a “lower threshold for ordering additional investigations.” The majority of the interviewed experts also stated that they categorise people that present with dryness-only symptoms versus people with systemic symptoms/organ involvement.

During the workshop, some clinicians described a younger subpopulation who have few symptoms of dryness but have signs of organ damage/disease activity. This subpopulation could be similar to the B-cell active disease and low symptom burden subpopulation explored by Nguyen et al. (2024) [20].

### Disease severity

During the interviews, some clinicians suggested that there were three severity levels for SjD: mild, moderate and severe. Working definitions for each severity level are presented in the ‘Suggested Definitions’ section of the Results. The clinicians agreed that before these definitions can be further refined, consensus is needed on what severity refers to – whether this is severity of disease activity, the nature of disease manifestations, the potential for irreversible damage, or the severity of symptoms. Some experts were opposed to combining disease severity and disease activity because they view them as distinct concepts.

There were conflicting opinions about whether people with mild SjD would have no prescribed medication. Some of the participating clinicians commented that people could still be classed as ‘mild’ if they were prescribed glucocorticoids. One clinician explained that an absence of prescribed medication does not necessarily indicate mild disease. For example, there may be no suitable medications for such a patient; this provides more of a commentary on the lack of SjD-specific therapies rather than the disease severity level.

The participating clinicians reported various methods for assessing disease severity. For example, this could include an evaluation of symptoms (including symptom burden and effect on the patient’s daily life) and/or system or organ involvement. This assessment may also be based on clinical observations and test results, with specific tests depending on the disease manifestation.

Clinicians and patients may also have a different understanding of severity. For example: “[…] patients may have severe symptoms but no systemic involvement. The disease is severe from the patient’s perspective, but clinicians don’t often consider a disease without systemic involvement to be severe (even though dryness might be presenting a serious burden to the patient).”

### Disease progression

Two aspects of disease progression were discussed in the interviews: progression in damage and progression in severity. While there are no internationally validated tools to assess damage, a damage index has been created in both Italy and the UK with limited use [21, 22]. To quantify disease progression, it is essential to document test results over time. However, several of the participating clinicians stated that there is an absence of a specific tool or formal process to measure disease progression. One clinician highlighted that this makes assessing disease progression very difficult because you need to individually assess each organ.

Overall, the interviewed clinicians gave numerous examples of tests that are used (routinely or less frequently) to assess disease progression. These included observations, blood tests (ESR, IgG, blood count, complement levels, cryoglobulinaemia), organ-specific tests (change in CT, lung function, urine tests, renal function), and dryness/glandular function tests (patient reported, salivary flow test, Schirmer test, glandular biopsies, glandular ultrasound).

The participating clinicians agreed that patients may have different priorities compared to physicians; for example, progression in symptoms may be more important from a patient perspective. While a working definition for disease progression is presented in the ‘Suggested Definitions’ section of the Results, including an element of patient empowerment in a future definition would be important.

### Disease remission

Overall, there was a high degree of uncertainty regarding the possibility of remission in SjD. Three of the interviewed clinicians noted that remission could be possible with the development of new treatments. Another stated that the only time they observe “remission” is where SjD has been incorrectly diagnosed.

The participating clinicians identified four key themes that could be considered in a future definition of remission: symptoms (no symptom burden versus low symptom burden), medication (no prescribed medication required versus low doses of certain medicines required), inflammation (no inflammation versus mild inflammation), and laboratory parameters (normal laboratory parameters versus slightly out-of-range parameters).

One of the clinicians expressed that discussions of disease remission are “very ambitious and potentially premature” given the many unknowns in SjD. However, they noted that these discussions are important given that remission is ultimately the target goal for patients. It is clear that more research, registry and longitudinal data are needed to inform future discussions regarding remission. As such, a working definition of remission was not prepared.

### Unmet needs

Throughout the SHAPE project, the clinicians agreed that “improved treatment options” are a major unmet need in SjD. Some of the participating clinicians discussed the need for specific treatments, such as those that prevent disease progression or lymphoproliferative complications and those that treat fatigue, pain, or neuropathies. Other unmet needs included improved diagnosis and referral for people with SjD.

### Suggested definitions

The working definitions for key clinical terms, prepared with clinician input throughout the SHAPE project, are presented in Table 1.

**Table 1:** Working definitions for key terms in SjD

Key term	Suggested definition
Active disease	Active SjD describes a state where a person experiences signs, symptoms and/or objective indications of inflammation or organ involvement associated with SjD. As SjD can affect a wide range of organs and systems, each organ should be individually assessed to determine if there is active disease. While the ESSDAI has some limitations in clinical practice (e.g. some relevant domains are not covered or are difficult to measure), it can be a valuable tool for identifying active SjD and/or guiding the selection of tests to assess disease activity within specific organs.
Mild SjD	Mild SjD describes where there is no or minimal systemic or organ involvement. People with mild SjD may have dryness or fatigue, but they manage symptoms well (have a good quality of life) and are not on prescribed medication.
Moderate SjD	Moderate SjD describes where patients experience constitutional symptoms or may present with laboratory abnormalities, arthralgia, joint symptoms, and glandular involvement/swelling. People with moderate SjD may require additional support to manage their disease, such as prescribed medication or replacement therapy, and the disease affects their quality of life.
Severe SjD	Severe SjD describes where there is organ involvement (and possibly irreversible organ damage) or where the patient has (at least) a high symptom burden. People with severe SjD are on treatment and may require more aggressive treatment options, such as immunosuppressive therapy. They may also require more frequent infusions, blood tests or monitoring. The disease affects the patient’s quality of life.
Disease progression	Progression involves uncontrolled disease activity that leads to organ damage or functional impairment. Progression can be either a continuous outcome (e.g. a decline in a single manifestation) or a categorical outcome (e.g. an expansion of different manifestations). Patients may have different priorities to clinicians. For example, progression in symptoms may be more important from a patient’s perspective, whereas progression of systemic disease activity may be more important from a clinician’s point of view. Disease progression is a multidimensional concept. For example, progression in damage describes where damage associated with SjD becomes worse over time.

Abbreviations: ESSDAI = EULAR Sjögren’s syndrome disease activity index, SjD = Sjögren’s disease

Notes: The working definitions are intended to promote discussion in the field, as opposed to being definitive and rigid. They were drafted based on discussions in the individual interviews and were presented to all clinicians during the group workshop – where they were invited to make amendments

## Discussion

There is high variability in international SjD clinical practice and expert opinion, likely because the disease itself is very heterogeneous. This makes it difficult to achieve consensus on key definitions at this stage. Therefore, this study hoped to form a starting point for future discussions and further collaboration in the field.

The working definitions suggested in this study have been prepared using an iterative process, incorporating feedback from experienced rheumatologists from several countries and focusing on clinical practice. The findings of this study are not intended to replace established and validated tools in clinical research. Instead, the definitions represent initial working concepts and are primarily intended to highlight key aspects that clinicians deem important in daily; the definitions are not validated and should be refined with further work.

One area that would benefit from future research is the concept of disease severity in SjD. While some of the participating clinicians suggested that there are three severity levels (mild, moderate, severe), there is not yet agreement on what ‘severity’ constitutes. It could range from the severity of disease activity, the nature of disease manifestations, the potential for irreversible damage, or the severity of symptoms.

Furthermore, there was no clear trend in the way that the interviewed clinicians define and evaluate disease activity in clinical practice, with disease indexes being inconsistently used during follow-up visits and appointments. It was unclear whether the patient perspective or impact on quality of life should be considered when defining active disease.

The participating clinicians also expressed that future iterations of clinical definitions should include how the term can be objectively measured in clinical practice (e.g. the tests/results that should be used to identify someone as having mild SjD). This would also help to reduce the variability between clinicians that is currently observed.

Further research should be conducted to build on the results presented and to explore the key themes highlighted in more depth. Specifically, qualitative research designs and Delphi panels would be recommended to ensure that any findings provide the most value for clinical practice. Moreover, the working definitions proposed in in this study do not incorporate patient perspectives. Once more consensus is reached from a clinical perspective, future research should also explore how patient perspectives can be incorporated to validate and expand the definitions.

Finally, future research should focus on developing new treatments for SjD, an area that all clinicians identified as a major unmet need. This is currently an active area of research, with more than 5 ongoing Phase 3, multicentre, randomised controlled trials [23–28].

### Strengths and limitations

The study used an iterative method and aligned with best practice recommendations for qualitative research. The clinicians who participated in the SHAPE project also have extensive experience and were recruited from several countries, providing different perspectives on disease management in daily practice. All participating clinicians were rheumatologists. However, as people with SjD require multidisciplinary care, it could be interesting to explore the opinion of specialists from other, related fields. The study was also limited by a small sample size, and a larger sample may have provided further insights. Moreover, the clinicians were recruited from a shortlist compiled by the study’s sponsor.

During the workshop, the clinicians were presented with draft definitions based on the individual interview discussions. To measure clinician agreement with each definition, interactive polls were used within the videoconferencing software. Any suggested amendments to the suggested definitions were discussed orally and reviewed in the ‘minutes’ document. However, formal agreement on the revised definitions (e.g. via a 5-point Likert scale) was not measured.

## Conclusion

SjD is a systemic disease with major unmet needs (primarily, an absence of treatment options). There is a lack of definitions specifically intended for daily clinical practice in SjD, which may contribute to the high variability in clinical assessment and management observed in this study. The results of this study are intended to guide future collaboration in the field, acting as an initial step to promote further discussion among SjD experts. Future discussions will also help to achieve consensus on key definitions and should particularly focus on the topics of disease severity and activity.

## Supplementary Information

Below is the link to the electronic supplementary material.

Supplementary Material 1

## Data Availability

No datasets were generated or analysed during the current study.

## Abbreviations

clinESSDAI: Clinical ESSDAI
ESSDAI: EULAR Sjögren’s Syndrome Disease Activity Index
ESSPRI: EULAR Sjögren’s Syndrome Patient Reported Index
ESR: Erythrocyte sedimentation rate
EULAR: European Alliance of Associations for Rheumatology
EQ-5D: EuroQol 5 Dimensions
SF-36: Short-Form 36
SHAPE: Sjögren’s Hands-On Practice Exchange
SjD: Sjögren’s disease
UK: United Kingdom
