# Supplementary material for: Sjögren’s Hands-On Practice Exchange (SHAPE): a qualitative, expert opinion project in Sjögren’s disease clinical practice
Source: BMC Rheumatol. 2025 Dec 20;10:4. doi: 10.1186/s41927-025-00606-8 (PMC12781536; doi:10.1186/s41927-025-00606-8)
Supplement: Supplementary file 1 — Supplementary Material 1 [file 41927_2025_606_MOESM1_ESM.pdf]

York Health Economics Consortium

SHAPE: Sjögren’s Hands-On Practice Exchange Project

Final Information Sheet and Consent Form Version 2.0

30/09/2024

Investors in People | Health & Wellbeing Award

UNIVERSITY of York

Information Sheet

1. Project Title

SHAPE: Sjögren’s **Ha**nds-On **P**ractice **E**xchange Project

2. Introduction

Sjögren’s disease (SjD) is a systemic, chronic autoimmune disease that affects glands, impairing their function and limiting the production of fluids. The most common symptoms are dry eyes, dry mouth, dry skin, swollen salivary glands, fatigue, and muscle pain. SjD affects a wide range of organs and systems, and symptoms may worsen over time. Current treatment focuses on symptom management rather than treating the cause of the disease, and a consensus on managing SjD and important disease definitions in clinical practice has not yet been established.

Novartis’s Medical Affairs Department has commissioned York Health Economics Consortium (YHEC) to undertake this expert opinion project in SjD. The project will facilitate a reflection exercise and conversation between clinicians to identify the commonalities and divergences in international clinical practice and to discuss potential practical approaches. This project is not aiming to define or validate any new clinical trial outcomes or tools, nor to produce clinical guidelines, which are being explored in other initiatives. Although commissioned by Novartis, the SHAPE project will not evaluate any specific products or treatments. Instead, the project will be oriented around current challenges in day-to-day clinical practice in SjD and will not be commercially focused.

Novartis has compiled a longlist of potential participants that may be eligible for this research, based on their clinical expertise in diagnosing and managing SjD. The list of potential participants was compiled from information available in the public domain.

We are inviting you to take part in a one-to-one interview and group workshop (both in English) as part of this project. Any work that you complete for this project will be reimbursed (see further information in Section 7). To be eligible for the project, you must:

1. Have expertise in the diagnosis and management of SjD.
2. Be based in the UK, Germany, Italy, France, Spain, the Netherlands, the USA, Canada, China or Japan.
3. Be fluent in English because the output from this work will be developed from the interviews and workshop.

Please see some further information about the project below.

3. Purpose

The vision of this project is to facilitate a conversation between clinicians around the similarities and differences in international clinical practice and to reach some alignment and consensus on potential practical approaches for disease management and other key concepts. To achieve this vision, the objectives of the project are:

- To gather clinician insights through one-to-one interviews followed by a group workshop.
- To discuss current challenges in clinical practice and to suggest potential practical approaches for evaluating disease activity, disease severity, classifying patients, and establishing treatment goals in SjD.
- To share the key learnings and experiences from the project with a global audience by submitting a peer-reviewed manuscript. This would allow any insights and practical approaches to be used by rheumatologists or healthcare professionals worldwide to help optimise care for people with SjD.

Ethical approval has been obtained from the Health Sciences Research Governance Committee at the University of York. If you decide to participate in this research, you will be asked to sign a consent form. You can withdraw from this study, including leaving the workshop, at any time, without requiring a reason and without any repercussions. If you decide to withdraw after your interview or the workshop has been completed, your data (including contributions to the workshop) will still be included in the study.

4. Interview Structure

In the first part of this project, you will be asked to attend a videoconference interview that will be scheduled for 1 hour in **October 2024**. We will organise the interview around your availability. The interview will be conducted using Zoom. The audio will be recorded in Zoom during the interview. Two researchers from YHEC will conduct the interview; one will lead the interview while the other takes notes.

The interview audio recording will be saved in the interview host's Zoom cloud. We will export and download a copy of the audio recording, which will be used to produce a summary of the interview and to check understanding of key points. All the recordings will be pseudonymised, and your name will not be saved in the recording or interview summary.

We will send you a summary of your interview to check for accuracy, which will take approximately 30 minutes. The summary will also be shared with Novartis. No transcripts of the interviews will be produced.

During the interview you should not disclose any additional personal information (other than information about your role as mentioned earlier) or any personal information of your patients. If there is any accidental disclosure of confidential information, this will not be transcribed and will be deleted from the audio recording.

Once all the interviews have been conducted, we will complete a thematic analysis (where common themes across the interviews are extracted from the interview summaries). Once themes

are extracted and agreed, we will conduct a qualitative synthesis of the themes and will summarise them into a slide deck or report, which will be sent to Novartis.

5. Workshop Structure

After the interview results are finalised, you will be invited to attend a videoconference workshop to validate the interview results and discuss potential practical approaches on evaluating disease activity, disease severity, classifying patients, and establishing treatment goals in SjD.

The workshop structure and content will be based on the outputs of the interviews, with key themes from the interviews informing the discussion points. The workshop attendees will include the other seven interviewed clinicians, YHEC experts (who will facilitate the workshop), and two representatives from Novartis (who will observe the workshop). The workshop will be conducted using Zoom and will be scheduled for 3 hours in early **December 2024**. The audio and video during the workshop will be recorded in Zoom.

After the workshop, we will prepare detailed minutes of the discussions and key points, which you will be asked to check for accuracy. This should take approximately 30 minutes. The workshop minutes will also be shared with Novartis. Due to their presence at the workshop, Novartis representatives will be able to identify individual contributions; however, any workshop quotes used in future scientific publications will be pseudonymised.

6. Dissemination

The results of this work (both interviews and workshop) may be disseminated via scientific publications (e.g. through conference abstracts, presentations and/or journal articles).

We might include pseudonymised quotes from your interview and/or the workshop in the slide deck / report to illustrate the themes and provide evidence of the main points of interest. Pseudonymised quotes may also be included in any scientific publications.

7. Honorarium

You will receive an honorarium of [£X per hour] (based on fair market value in your country) for participating in this project. In total, you will be reimbursed for 5 hours of work. This includes time to attend the interview, check the interview summary for accuracy, attend the workshop, and check the workshop minutes for accuracy.

The contracting will be between YHEC and yourself (or your institution, if required). You will receive payment after you have reviewed the workshop minutes.

8. Adverse Event Reporting

As this research has been commissioned by a pharmaceutical company, we are required to pass on any adverse events / product complaints pertaining to their products that are mentioned during the interview or workshop. If this happens, we will need to collect details and report the adverse event, even if you have already reported this. You will be asked whether you consent to YHEC passing on your name and contact details to the company’s drug safety department for their follow up, but you may choose to remain anonymous.

9. Data Protection

You will be assigned a unique code which will be used throughout the project. Data will never be presented with real names (except the consent forms).

All data (identification data, consent forms, interview summaries, and recordings) will be stored in password protected folders on a password protected computer. These data files will only be accessible to the YHEC researchers working on this project. Novartis will not be provided with the consent forms or interview / workshop recordings. At the end of the project, the pseudonymised summaries of the interviews of consenting interviewees will be sent to Novartis.

All data will be securely retained for a period of six years and will be disposed of appropriately after this period. The data will be added to the YHEC Data Register that is used to keep a record of datasets containing sensitive and personal data. The register includes how long the data need retaining for, a date by which the data should be deleted, and confirmation that the data have been deleted by the person responsible for this.

The YHEC privacy policy is available here: YHEC Privacy Policy. As explained in Section 4 of the privacy policy, you have a number of rights in relation to your personal data. These include the right to:

- Find out how we process your personal data.
- Request that your personal data is corrected if you believe it is incorrect or inaccurate.
- Withdraw your consent to our processing of your personal data.
- Obtain a copy of the personal information that we hold about you. We will take steps to verify your identity before responding to your request and will respond as soon as possible (after receipt of confirmation of your identify) and in any event within one month.

If you would like to exercise any of your rights outlined in this policy or have any questions about the way in which YHEC handles your personal data, please contact us in writing at yhec@york.ac.uk.

10. Contact Details

If you have any further questions or require more information, please contact:

Dr Emily Gregg

emily.gregg@york.ac.uk

If you would like independent advice or to make a general complaint, please contact the YHEC Director:

Professor Matthew Taylor

matthew.taylor@york.ac.uk

If you would like to make a data-related complaint, please contact:

The University of York's Data Protection Officer

dataprotection@york.ac.uk

The Information Commissioner's Office

www.ico.org.uk/concerns

Consent Form

Please complete and return to emily.gregg@york.ac.uk. Please put your **initials** in the empty boxes if you agree with the following statements and answer 'Yes' or 'No' to the two questions.

I confirm that I have read the information sheet (Version 2.0, dated 30.09.24).	
I confirm that I am happy to participate in this project including an interview and workshop.	
I confirm that I am happy for the interview and workshop to be recorded.	
I confirm that I am happy for the project results and pseudonymised quotes from the interview/workshop to be included in the project report / slide deck and any future scientific publications.	
I confirm that I am happy for YHEC to contact me regarding the accuracy of the interview summary and the workshop minutes.	
I confirm that I am happy for a pseudonymised summary of the interview and the workshop minutes to be sent to Novartis.	
I understand that if I mention any relevant adverse events / product complaints during the interview or workshop, this information will be passed to Novartis.	
If I mention any relevant adverse events / product complaints, I consent to my name and contact details being shared with Novartis's drug safety department for their follow up.	
Would you like to receive a copy of any future scientific publications of this research (e.g. conference abstracts/posters or journal articles, if publicly available)?	Yes / No

Are you happy to be contacted by YHEC in the future about other relevant research projects?	Yes / No
**Signature:**	
**Name:**	
**Date:**	

York Health Economics Consortium

NOVARTIS

SHAPE: Sjögren’s Hands-On Practice Exchange Project

Final Topic Guide for Interviews

EMILY GREGG, Senior Research Consultant

RACHAEL McCOOL, Associate Director

CHARLOTTE GRAHAM, Research Consultant

DEBORAH WATKINS, Research Consultant

KAZ BARTLETT, Research Assistant

02/10/2024

INVESTORS IN PEOPLE
We invest in people Gold

UNIVERSITY of York

1 Introduction

Estimated time to complete this section: 5 minutes.

- Thank the interviewee for agreeing to take part.
- Give a brief introduction to the York Health Economics Consortium (YHEC) and its role in the project:
  - YHEC is a health economic consulting company owned by the University of York. YHEC provides national and international consultancy in health economics and outcomes research to the NHS, pharmaceutical and healthcare industries.
  - As explained in the information sheet, Novartis has commissioned YHEC to undertake this project called **SHAPE**: **S**jögren's **Ha**nds-On **P**ractice **E**xchange. The project aims to facilitate a conversation between clinicians around the similarities and differences in international clinical practice for Sjögren's disease (SjD) and to reach some alignment and consensus on potential practical approaches for disease management and other key concepts.
  - The first stage includes the one-to-one interviews, which will be followed by a group workshop. The final objective is to share the key learnings and experiences from the project with a global audience by submitting a peer-reviewed manuscript.
- Remind the interviewee that:
  - The interview is scheduled for 60 minutes and will be recorded (as per the consent form).
  - They should not disclose any personal information or confidential patient information during the interview (other than when asked about their role / previous experience).
  - Any accidental disclosure of confidential information will not be transcribed and will be deleted from the audio recording.
  - Novartis must keep records of any side effects or complaints that people may have about their products, and we must assist the company in meeting its legal obligations. Therefore, if, during the interview, there is any reference made to a side effect or complaint about a medicine, we will let the company know about this even if it has already been reported directly to the company or the regulatory authorities.
- Explain that we will begin the interview with some questions about their role/experience before asking questions about the classification of SjD, clinical assessment of disease progression and severity, and treatment decisions in SjD. Remind the interviewee that we are interested in feedback based on their **day-to-day clinical practice**.
- Check if the interviewee has any questions before starting the interview.
- **Start the recording in Zoom**.

2 Classification of SjD in Clinical Practice

Estimated time to complete all questions in this section: 15 minutes.

2.1 How do you classify SjD in your clinical practice?

- **Prompt 1:** Do you still classify SjD into ‘primary’ and ‘secondary’ disease, or is this being phased out / becoming obsolete in clinical practice?
- **Prompt 2:** Do you systematically apply the ACR-EULAR classification criteria in your practice?
  - **N.B: A ‘yes / no’ answer would suffice for this question and does not need to be elaborated on.**
  - **N.B: Examples of other classification criteria that have been produced are listed in Table 1.**

2.2 Beyond the conventional classification of ‘primary’ and ‘secondary’ disease, are there any subpopulations identified in real-life practice in SjD?

- **N.B: The phrasing of this question should be guided by the response to question 3.2.**
- **Prompt 1:** This could be based on the presence of:
  - Systemic manifestations / organ involvement (dryness-only symptoms vs systemic / organ involvement).
  - Severity levels (mild, moderate, severe).
  - Disease activity levels (inactive, mildly active, very active).
  - Rate of progression (e.g. rapid progression).
  - Age of onset (e.g. early SjD).
  - The presence of glandular involvement (glandular vs extraglandular).
  - The balance of glandular / extraglandular symptoms (glandular predominant vs extraglandular predominant) [1].
- **Prompt 2:** The ACR-EULAR classification introduced additional criteria around glandular manifestations of SjD. Do you classify people according to glandular-dominant vs extraglandular-dominant disease activity? Do you think it is important to focus on the systemic / organ manifestations of the disease when classifying SjD?
- **Prompt 3:** A study by Nguyen et al. (2024) identified three patient subgroups: those with B-cell active disease and low symptom burden (BALS), those with high systemic disease activity (HAS), and those with low systemic disease activity and high symptom burden (LSAHS). Does this reflect the subpopulations you see in clinical practice [4]?

- **Prompt 4:** B-cell lymphoma is a major complication of SjD. Would you classify patients based on their risk of developing lymphoma [2, 3]?

2.3 What are the main unmet needs for each subpopulation you have mentioned?

2.4 Do you consider SjD as a primarily B-cell driven condition? Does this impact your therapeutic approach?

- **N.B: A 'yes / no' answer would suffice for this question and does not need to be elaborated on.**

Table 1: Different SjD classification criteria since 2000

Classification system	Key features of criteria
2002 AECG classification criteria [5]	i. Ocular symptoms<br>ii. Oral symptoms<br>iii. Ocular signs (Schirner's test, van Bijsterveld score)<br>iv. Histopathology<br>v. Objective salivary gland involvement (salivary flow, sialography, scintigraphy)<br>vi. Autoantibodies (Ro/SSA or La/SSB)<br>≥4 criteria as long as either (iv) or (vi) are positive<br>**Autoantibodies and/or histopathology mandatory**
2012 ACR classification criteria [6]	i. Autoantibodies: (Ro/SSA and/or La/SSB) or (RF and ANA ≥1:320)<br>ii. Histopathology<br>iii. Ocular staining score ≥3<br>≥2 criteria<br>**Autoantibodies and/or histopathology mandatory**
2016 ACR-EULAR classification criteria [7]	i. Histopathology (3 points)<br>ii. Ro/SSA (3 points)<br>iii. Ocular staining score ≥5 (or van Bijsterveld score ≥4) (1 point)<br>iv. Schirmer's test ≤5 mm/5 min in at least one eye (1 point)<br>v. Unstimulated whole saliva flow rate ≤0.1 ml/min (1 point)<br>≥4 points<br>**Autoantibodies and/or histopathology mandatory**

Key: ACR - American College of Rheumatology; AECG - American-European Consensus Group; ANA - antinuclear antibody test; EULAR - European Alliance of Associations for Rheumatology; RF - rheumatoid factor.

3 Clinical Assessment of SjD Progression, Disease Activity, and Severity

Estimated time to complete all questions in this section: 10 minutes.

3.1 How often are people with SjD assessed?

- **Prompt 1:** Is there a specific period after which patients are reassessed, or do you wait for patients to re-present with new/worsened symptoms?
- **Prompt 2:** Are patients regularly seen by a clinician for a full assessment, or are they reviewed for different symptoms at different times/intervals?

3.2 In other diseases, there are clearly defined stages of disease progression. How do you **assess** progression in SjD in clinical practice?

- **Prompt 1:** Do you use clinical scores, observations, or other factors to define progression? This could include assessment of clinical symptoms, a Schirmer test, worsening of salivary flow, progression from glandular to extraglandular symptoms.

3.3 How do you **define** progression in SjD in clinical practice?

3.4 How do you define active disease in your clinical practice?

3.5 Can patients have active disease that is not progressive in SjD?

3.6 Can patients have progressive disease without disease activity?

- **Prompt 1:** For example, is the disease progressing when patient's symptoms are worsening (as measured by ESSPRI), but there is no change in disease activity, which is low (as measured by ESSDAI)?

3.7 There are a number of indexes that can be used to assess disease activity and patient symptoms in SjD. These include the ESSDAI, ClinESSDAI, ESSPRI, CRESS, and STAR indexes (Table 2). How useful are these indexes (and their scores) in clinical practice?

- **Prompt 1:** Do you use these scores systematically with all patients? If yes, do you complete them via an app, on a computer, or using paper?
- **Prompt 2:** If you don't use these scores in clinical practice, do you use the elements of these indexes to assess disease activity (i.e. asking the index questions without officially recording the score)?
- **Prompt 3:** Do you use any other indexes or measures to assess disease activity and patient symptoms?

3.8 Which parameters do you use to measure the severity of SjD?

- **Prompt 1:** Is this based on scores (Table 2), clinical guidelines (Table 3), and/or symptoms?

3.9 How do you define ‘mild, ‘moderate’ and ‘severe’ SjD?

Table 2: Summary of SjD indexes

Index	Purpose of index	What does the tool involve?
**ESSDAI (2010):** EULAR Sjögren's Syndrome Disease Activity Index [8]	Clinician-reported outcome measure. It is now the gold standard in clinical trials of primary SjD.	▪ It involves 12 domains: constitutional, lymphadenopathy, glandular, articular, cutaneous, pulmonary, renal, muscular, central nervous system, peripheral nervous system, haematological, biological.<br>▪ Each domain is divided into 3 to 4 levels of activity.<br>▪ Each domain is scored based on the severity of clinical findings, and the total ESSDAI score is used to gauge overall disease activity.<br>▪ It also includes organ-by-organ definitions that were agreed by a large number of experts.
**ESSPRI (2011):** EULAR Sjögren's Syndrome Patient Reported Index [9]	Patient-reported outcome measure. It assesses the severity of patient symptoms (e.g. pain, fatigue, dryness) in the previous 2 weeks.	▪ A 3-item patient-reported outcome measure: dryness, limb pain and fatigue.<br>▪ Items are rated on a 0 to 10 numerical rating scale, and a mean score is calculated. Higher scores indicate greater symptom severity.
**ClinESSDAI (2016):** Clinical ESSDAI [8]	The ‘biological’ domain in the original ESSDAI may falsely increase association between a new biomarker and the true clinical activity.	▪ The same domains as ESSDAI but excluding the ‘biological’ domain.
**CRESS (2021):** Composite of Relevant Endpoints for Sjögren's Syndrome [10]	A composite endpoint including multiple existing clinically relevant measures for assessing treatment efficacy in primary SjD.	▪ Systemic disease activity (ClinESSDAI <5).<br>▪ Patient-reported symptoms (ESSPRI decrease ≥1 point and/or ≥15%).<br>▪ Tear gland (in patients with abnormal Schirmer or ocular staining score at baseline an increase ≥5 mm or decrease ≥2 points, respectively. In patients with normal values no change to abnormal for either).<br>▪ Salivary gland (unstimulated whole saliva secretion increase ≥25% or salivary gland ultrasonography decrease ≥25%).<br>▪ Serological (rheumatoid factor decrease ≥25% or immunoglobulin G decrease ≥10%).
**STAR (2022):** Sjögren Tool for Assessing Response [11]	Recent RCTs showed that improvement in ESSDAI does not necessarily translate to improvement in ESSPRI. Thus, ESSDAI does not capture all important disease features when used as a primary endpoint. STAR was developed to resolve this issue and is intended for use in clinical trials as an efficacy endpoint [11].	▪ Five core domains: systemic activity, patient symptoms, lachrymal gland function, salivary gland function, biological parameters.<br>▪ Assesses response to treatments on all disease aspects in clinical trials.

Key: ESSDAI - EULAR Sjögren's Syndrome Disease Activity Index; ESSPRI - EULAR Sjögren's Syndrome Patient Reported Index; EULAR - European Alliance of Associations for Rheumatology; RCT - randomised controlled trial; RF - rheumatoid factor; SjD - Sjögren's Disease; STAR - Sjögren Tool for Assessing Response.

Table 3: Summary of SjD clinical guidelines

Clinical guidelines	Details
2018 Sjögren's Syndrome Foundation treatment guidelines [12]	▪ For musculoskeletal pain, first line treatment is hydroxychloroquine (HCQ), second-line treatment is methotrexate (MTX), and third-line treatment is HCQ + MTX.<br>▪ The use of HCQ to treat fatigue should only be considered in "select situations"; there is no guidance on what this constitutes.<br>▪ **Biologics:** TNF-alpha inhibitors are not recommended in primary SjD; rituximab may be considered.<br>▪ Each recommendation is ranked 'strong', 'moderate' or 'weak', depending on the strength of the evidence base.<br>▪ Clinicians are "advised to consider an individual patient's circumstances when weighing the risks and benefits of each therapy".
2019 EULAR recommendations [13]	▪ Treatment goals appear to be symptom management. The first therapeutic approach for dryness should be symptomatic relief using topical therapies.<br>▪ Systemic therapies may be considered where active systemic disease is ≥1 ClinESSDAI score.<br>▪ Therapeutic response is defined as a decrease of ≥3 points in the global ESSDAI score.
2024 British Society for Rheumatology guidelines for disease management [14]	Guidance is presented in four steps:<br>▪ **Step 1:** Confirm diagnosis (in line with the 2016 ACR/EULAR criteria).<br>▪ **Step 2:** Treat symptoms (advice for treatment of dry eyes, dry mouth, and systemic dryness).<br>▪ **Step 3:** Systemic management (consider HCQ and other DMARDs for specific indications).<br>▪ **Step 4:** Extras and special situations (e.g. pregnancy, co-morbidities and lymphoma).

Key: ACR - American College of Rheumatology; EULAR - European Alliance of Associations for Rheumatology; HCQ - hydroxychloroquine; MTX - methotrexate; TNF-alpha - tumour necrosis factor alpha.

4 SjD Treatment Decisions

Estimated time to complete all questions in this section: 25 minutes.

5.1 What is the current treatment goal in SjD, and how is this defined in clinical practice?

- **Prompt 1:** How should this be measured in clinical practice?
- **Prompt 2:** How should this be communicated to patients?

5.2 What should the treatment goal be for a Sjögren's specific drug (i.e. a targeted therapy)?

- **Prompt 1:** Should the treatment goal change depending on the population?

5.3 What factors influence treatment choice in SjD?

- **Prompt 1:** How do you decide which treatment(s) to use? The treatments of interest are hydroxychloroquine, systemic glucocorticoids, and disease-modifying antirheumatic drugs (DMARDs, even if used off label).
- **Prompt 2:** Is your decision informed by any tools or guidelines (if so, please provide a reference)? For example, do you use the 2018 guidelines developed by the Sjögren's Syndrome Foundation [12], the EULAR 2020 recommendations [13], or the 2024 British Society for Rheumatology (BSR) guidelines for disease management [14] (Table 3)?
  - **Note:** Interested in treatments that are impacting the mechanism of SjD. The BSR guidelines recommend hydroxychloroquine to be considered in those with significant fatigue and systemic symptoms. EULAR says glucocorticoids should be used at the minimum dose and length of time necessary to control active systemic disease. Neither guideline mentions DMARDs.
- **Prompt 3:** Is your treatment choice guided by the subpopulations you observe in clinical practice?

5.4 How do you decide if a treatment is working and when to start / stop treatment? Does this vary depending on the treatment used?

- **Prompt 1:** The EULAR 2020 guidelines define 'therapeutic response' as decrease of ≥3 points in the global ESSDAI score [13]. Do you use this when defining response in people with SjD?

5.5 Is remission likely, or even possible, in SjD?

- **Prompt 1:** How do / would you define remission in SjD? For example, is this based on the absence of symptoms for a specific period of time. Do you assess remission using any specific tests or tools?
- **Prompt 2:** We note that 'complete disease remission' is not possible in patients with "damage to gland architecture" as reported by the National Academies of Sciences, Engineering, and Medicine [15]. Are there other patients in whom 'complete disease remission' is not possible?
- **Prompt 3:** What about 'partial remission'? How do 'partial' and 'complete' remission differ?

5.6 How do you determine if SjD is active or controlled in practice?

5.7 Are flares relevant in SjD? If so, how is a flare defined and managed? Does a flare have a limited duration? When and how is clinical intervention decided after a flare?

- **Prompt 1:** 7 members of the patient advocacy group involved in the European NECESSITY project have published the following definition: “A flare is the temporary and sudden onset of symptoms that become significantly worse before resolving and, on average, last at least 3 days” [16]. Do you agree with this definition?

- **Prompt 2:** Do you use any specific tools when assessing or managing flares in SjD?

5.8 Disease-modifying therapies are treatments that can help change the progression of SjD over time. Do you think there are currently any disease-modifying treatments for SjD?

5.9 Should biologics be used in SjD? If so, in what population? **When** should biologics be used?

- **Prompt 1:** We note that, according to the British Society for Rheumatology 2024 guidelines, biologics are not approved by the National Institute for Health and Care Excellence for SjD [14]. Whereas the 2020 EULAR recommendations state that biologics should be restricted to patients with ‘active systemic disease (ClinESSDAI score ≥1)’ [13].

5 Final Comments

Estimated time to complete this section: 5 minutes.

- Ask if the interviewee would like to add any final comments.
- Stop and save the recording in Zoom.
- Thank the interviewee for participating in the interview.

Remind the interviewee of the next steps:

- We will ask them to review a summary of their interview for accuracy.
- We will invite them to participate in the group workshop, with the other seven interviewed clinicians. We will be in touch to get their availability for this asap.

6 References

1. Gairy K, Knight C, Anthony P, Hoskin B. Burden of illness among subgroups of patients with primary Sjögren’s syndrome and systemic involvement. Rheumatology. 2020.60(4):1871-81. doi: 10.1093/rheumatology/keaa508

2. Barcelos F, Brás-Geraldes C, Martins C, Papoila A-L, Monteiro R, Cardigos J*, et al.* Added value of lymphocyte subpopulations in the classification of Sjögren's syndrome. Scientific Reports. 2023.13(1):6872. doi: 10.1038/s41598-023-31782-7

3. Hamza N, Bos NA, Kallenberg CGM. B-cell populations and sub-populations in Sjögren's syndrome. La Presse Médicale. 2012.41(9, Part 2):e475-e83. doi: https://doi.org/10.1016/j.lpm.2012.05.021

4. Nguyen Y, Nocturne G, Henry J, Ng W-F, Belkhir R, Desmoulins F*, et al.* Identification of distinct subgroups of Sjögren's disease by cluster analysis based on clinical and biological manifestations: data from the cross-sectional Paris-Saclay and the prospective ASSESS cohorts. The Lancet Rheumatology. 2024.6(4):e216-e25.

5. Vitali C, Bombardieri S, Jonsson R, Moutsopoulos HM, Alexander EL, Carsons SE*, et al.* Classification criteria for Sjögren's syndrome: a revised version of the European criteria proposed by the American-European Consensus Group. Annals of the rheumatic diseases. 2002.61(6):554-8. doi: 10.1136/ard.61.6.554

6. Shiboski SC, Shiboski CH, Criswell L, Baer A, Challacombe S, Lanfranchi H*, et al.* American College of Rheumatology classification criteria for Sjögren's syndrome: a data-driven, expert consensus approach in the Sjögren's International Collaborative Clinical Alliance cohort. Arthritis care & research. 2012.64(4):475-87. doi: 10.1002/acr.21591

7. Shiboski CH, Shiboski SC, Seror R, Criswell LA, Labetoulle M, Lietman TM*, et al.* 2016 American College of Rheumatology/European League Against Rheumatism classification criteria for primary Sjögren's syndrome: A consensus and data-driven methodology involving three international patient cohorts. Annals of the rheumatic diseases. 2017.76(1):9-16. doi: 10.1136/annrheumdis-2016-210571

8. Seror R, Bowman SJ, Brito-Zeron P, Theander E, Bootsma H, Tzioufas A*, et al.* EULAR Sjögren's Syndrome Disease Activity Index (ESSDAI): a user guide. RMD Open. 2015.1(1):e000022. doi: 10.1136/rmdopen-2014-000022

9. Seror R, Ravaud P, Mariette X, Bootsma H, Theander E, Hansen A*, et al.* EULAR Sjogren's Syndrome Patient Reported Index (ESSPRI): development of a consensus patient index for primary Sjogren's syndrome. Annals of the rheumatic diseases. 2011.70(6):968-72. doi: 10.1136/ard.2010.143743

10. Arends S, de Wolff L, van Nimwegen JF, Verstappen G, Vehof J, Bombardieri M*, et al.* Composite of Relevant Endpoints for Sjögren's Syndrome (CRESS): development and validation of a novel outcome measure. The Lancet. Rheumatology. 2021.3(8):e553-e62. doi: 10.1016/s2665-9913(21)00122-3

11. Seror R, Baron G, Camus M, Cornec D, Perrodeau E, Bowman SJ*, et al.* Development and preliminary validation of the Sjögren's Tool for Assessing Response (STAR): a consensual composite score for assessing treatment effect in primary Sjögren's syndrome. Annals of the rheumatic diseases. 2022.81(7):979-89. doi: 10.1136/annrheumdis-2021-222054

12. Vivino FB, Carsons SE, Foulks G, Daniels TE, Parke A, Brennan MT*, et al.* New treatment guidelines for Sjögren's disease. Rheumatic diseases clinics of North America. 2016.42(3):531-51. doi: 10.1016/j.rdc.2016.03.010

13. Ramos-Casals M, Brito-Zerón P, Bombardieri S, Bootsma H, De Vita S, Dörner T*, et al.* EULAR recommendations for the management of Sjögren’s syndrome with topical and

systemic therapies. Annals of the rheumatic diseases. 2020.79(1):3-18. doi: 10.1136/annrheumdis-2019-216114

14. Price EJ, Benjamin S, Bombardieri M, Bowman S, Carty S, Ciurtin C*, et al.* British Society for Rheumatology guideline on management of adult and juvenile onset Sjögren disease. Rheumatology (Oxford, England). 2024.doi: 10.1093/rheumatology/keae152

15. National Academies of Sciences Engineering and Medicine. Sjögren's Disease/Syndrome. In: Selected Immune Disorders and Disability. Washington DC: The National Academies Press; 2022.

16. Bouillot C, Hammitt KM, Lindland AH, Oosterbaan M, Pincemin M, Stone L. "Flare, did you say flare?" Flares ub Sjogren's disease: the patient perspective. Annals of the rheumatic diseases. 2023.82(Suppl 1):2103.
